# Localized Simple Multiple Kernel K-Means Clustering with Matrix-Induced Regularization

**DOI:** 10.1155/2023/6654304

**Published:** 2023-03-17

**Authors:** Jiaji Qiu, Huiying Xu, Xinzhong Zhu, Michael Adjeisah

**Affiliations:** ^1^School of Computer Science and Technology, Zhejiang Normal University, Jinhua 321004, China; ^2^AI Research Institute of Beijing Geekplus Technology Co. Ltd., Beijing 100101, China

## Abstract

Multikernel clustering achieves clustering of linearly inseparable data by applying a kernel method to samples in multiple views. A localized SimpleMKKM (LI-SimpleMKKM) algorithm has recently been proposed to perform min-max optimization in multikernel clustering where each instance is only required to be aligned with a certain proportion of the relatively close samples. The method has improved the reliability of clustering by focusing on the more closely paired samples and dropping the more distant ones. Although LI-SimpleMKKM achieves remarkable success in a wide range of applications, the method keeps the sum of the kernel weights unchanged. Thus, it restricts kernel weights and does not consider the correlation between the kernel matrices, especially between paired instances. To overcome such limitations, we propose adding a matrix-induced regularization to localized SimpleMKKM (LI-SimpleMKKM-MR). Our approach addresses the kernel weight restrictions with the regularization term and enhances the complementarity between base kernels. Thus, it does not limit kernel weights and fully considers the correlation between paired instances. Extensive experiments on several publicly available multikernel datasets show that our method performs better than its counterparts.

## 1. Introduction

Clustering is a widely used machine learning algorithm [1–4]. Multikernel clustering is one of the clustering methods which is based on multiview clustering and performs clustering by implicitly mapping sample points of different views to high dimensions. Many studies have been carried out in recent years [5–9]. For example, early work [10] shows that kernel matrices could encode different views or sources of the data, and MKKM [11] extends the kernel combination by adapting the weights of kernel matrices. Gönen and Margolin [12] improve the performance of MKKM by focusing on sample-specific weights on the correlations between neighbors to obtain a better clustering, called localized MKKM. Du et al. [13] engaged the *ℓ*_2,1_ norm to reduce the uncertainty of algorithm results due to unexpected factors such as outliers. To enhance the complementary nature of base kernels and reduce redundancy, Liu et al. [14] employed a regularization term containing a matrix that measures the correlation between base kernels to facilitate alignment. Other works [15–19]are different from the original MKKM method [11] that prefused multiple view kernels. These methods first obtain the clustering results of each kernel matrix, then fuse each clustering result in a later stage to obtain a unified result.

More recently, a newly proposed optimization strategy, simple multiple kernel k-means (SimpleMKKM) [20] has emerged as a representative of multikernel clustering (MKC). Different from the normal MKKM algorithm, SimpleMKKM assumes minimization of kernel weights and maximization of cluster partition, which leads to min-max optimization that is somewhat difficult to unravel. It converts the optimization to a minimization problem and cleverly solves it with a specially designed gradient descent method rather than a coordinate descent method. However, it is established that the strict alignment of the combined kernel matrix can force the combination globally. Therefore, Liu et al. proposed [21] localized SimpleMKKM, which reduces the negative impact of distant samples on clustering by restricting the kernel alignment to the k-nearest neighbors of the sample rather than the global alignment. In this way, LI-SimpleMKKM can sufficiently account for the variation between samples, improving clustering performance.

Although localized SimpleMKKM shows excellent performance on MKC problems, we find that the correlation between the given kernels is not sufficiently considered providing an opportunity for improvement based on the listed problem statement.

- The original method [21] makes the data stable by setting a larger weight *η*_*u*_ in the gradient descent step and maintaining the summation and nonnegativity of the weights through the association with other weights. However, this idea only enhances the correlation between different view weights and *η*_*u*_ and does not consider the relationship between view kernel matrices, especially between pairs.
- The original method is possible to select multikernel kernels with high correlation for clustering simultaneously. Repeated selection of similar information sources makes the algorithm redundant and has low diversity, leading to the low ratio of different kernel matrices' effectiveness, ultimately affecting the accuracy of the clustering results.

Motivated by these, we propose a localized SimpleMKKM with matrix-induced regularization (LI-SimpleMKKM-MR) to improve upon the LI-SMKKM algorithm by adding an entry containing a matrix to measure the correlation between each two basis kernel matrices. LI-SimpleMKKM-MR algorithm can reduce the probability and simultaneously select high-correlation kernels, thereby enhancing the diversity of synthetic kernels and enhancing the complementarity of low-correlation kernels. Moreover, it adopts the advantage of localized SimpleMKKM, which has a better optimization effect that can be achieved by clustering the neighbor index matrix formed by the sample and the nearest *k* neighbors, and uses the optimization strategy min_*η*_  − max_*H*_ instead of min_*η*_  − min_*H*_.

Compared with the original multiple kernel clustering, the proposed method optimizes kernel matrix weights by using gradient descent rather than coordinate descent, combined with localized sample alignment and kernel matrix induced regularization. This reduces the negative effects of forced alignment of long-distance samples and high redundancy and low complementarity of multiple kernel matrices.

We experimented with the algorithm on 6 benchmark datasets and compared it with the other nine baseline algorithms that solve similar problems through four indicators: clustering accuracy (ACC), normalized mutual information (NMI), purity, and rand index. We find that LI-SimpleMKKM-MR outperforms other methods. This is the first work to fully consider and solve the correlation problem between the base kernels to the best of our knowledge.

The contributions of this method are summarized as follows:

1. Proposed algorithm LI-SimpleMKKM-MR can productively deal with the alignment problem between kernel matrices using a regularization term, in order to reduce the redundancy, enhance the complementarity, and correlation between kernel matrices.
2. The novelty is that our proposed method can be transformed into SimpleMKKM or LI-SimpleMKKM by adjusting the hyperparameters, making LI-SimpleMKKM-MR an extension of the previous two methods.
3. We conducted extensive experiments on 6 public multiple kernel datasets using 4 metrics. The results show that our method achieves state-of-the-art performance compared to 9 existing baseline algorithms. The experiments essentially validate our understanding of the previous problems and the effectiveness of the proposed solution.

## 2. Related Works

### 2.1. Multiple Kernel K-Means

Let {**x**_*i*_}_*i*=1_^*n*^ ∈ *χ* be a set of *n* samples, and *ϕ*_*p*_(·) : **x** ∈ *χ*⇒*ℋ*_*p*_ means mapping the features of the sample **x** of the *p*th view into a high-dimensional Hilbert space *ℋ*_*p*_(1 ≤ *p* ≤ *m*). According to this theory, each sample can be represented by *ϕ*_*η*_(x)=[*η*_1_*ϕ*_1_^⊤^(x),…, *η*_*m*_*ϕ*_*m*_^⊤^(x)], where *η*=[*η*_1_, ⋯*η*_*m*_]^⊤^ means the weights of *m* prespecified base kernels {**K**_*p*_(·,·)}_*p*=1_^*m*^. The kernel weights will be changed according to the algorithm optimizing in the kernel learning step. According to the definition of *ϕ*_*η*_(x) and the definition of kernel function, the kernel function can be defined as follows:

(1) $$\begin{array}{c} K_{\eta }\left(x_{i},x_{j}\right)={\phi_{\eta }}^{⊤}\left(x_{i}\right)\phi_{\eta }\left(x_{j}\right)=\overset{m}{\underset{p=1}{\sum }}\eta_{p}^{2}K_{p}\left(x_{i},x_{j}\right)\text{.} \end{array}$$

We can use training samples {**x**_*i*_}_*i*=1_^*n*^ by (1) to calculate a kernel matrix **K**_*η*_. Based on the calculation of **K**_*η*_, the objective function of MKKM with **K**_*η*_ can be expressed as follows:

(2) $$\begin{array}{c} \begin{array}{c} \min_{H,\eta }Tr\left(K_{\eta }\left(I_{n}-{HH}^{⊤}\right)\right)s.t.\;H^{⊤}H=I_{k},\\ \eta^{⊤}1_{m}=1,\eta_{p}\geq 0,\forall p. \end{array} \end{array}$$

Here, **H** ∈ *ℝ*^*n*×*k*^ means one soft label matrix, which is used to solve NP-hard problems caused by the direct use of hard allocation, which is also called the partition matrix. Moreover, **I**_*k*_ means an identity matrix which is *k* × *k* in size.

Optimization of (2) can be divided into 2 steps: optimizing **H** or *η* and fixing the other one.

(i) Optimizing **H** with *η* is fixed, the problem of optimizing **H** in (2) can be represented as follows:
  (3) $$\begin{array}{c} \max_{H}Tr\left(H^{⊤}K_{\eta }H\right)s.t.\;H\in R^{n\times k}\text{,}\\ \begin{array}{c} {H^{⊤}H=I}_{k}. \end{array} \end{array}$$
The optimization of **H** of (3) can be easily solved by taking the first *k* eigenvalues of the matrix **K**_*η*_.
(ii) Optimizing *η* with **H** is fixed, with the soft label matrix **H** is fixed, the problem of optimizing *η* in (2) can be represented as follows:
  (4) $$\begin{array}{c} \begin{array}{c} \min_{\eta }\overset{m}{\underset{p=1}{\sum }}\eta_{p}^{2}Tr\left(K_{p}\left(I_{n}-{HH}^{⊤}\right)\right)s.t.\;H^{⊤}H=I_{k},\\ \begin{array}{c} \eta^{⊤}1_{m}=1,\eta_{p}\geq 0,\forall p \end{array}. \end{array} \end{array}$$
According to the constraints, it can be easily solved by the Lagrange multiplier method [10].

### 2.2. MKKM with Matrix-Induced Regularization

As (2) shows that *η*_*p*_ only depends on **K**_*p*_ and **H**. However, the interactions between different kernel matrices are not considered. Liu et al. [14] defined a criterion *ℳ*(**K**_*p*_, **K**_*q*_) to measure the correlation between **K**_*p*_ and **K**_*q*_. A larger *ℳ*(**K**_*p*_, **K**_*q*_) means high correlation between **K**_*p*_ and **K**_*q*_, and a smaller one implies that their correlation is low. By introducing the criterion term in (2), we can obtain the following objective function:

(5) $$\begin{array}{c} \begin{array}{c} \begin{array}{c} \underset{H,\eta }{\mathrm{Min}}Tr\left(K_{\eta }\left(I_{n}-{HH}^{⊤}\right)\right)+\frac{\lambda }{2}\eta^{⊤}M\eta \;s.t.\;H\in R^{n\times k},\\ {H^{⊤}H=I}_{k},\\ \eta^{⊤}1_{m}=1,\eta_{p}\geq 0,\forall p, \end{array} \end{array} \end{array}$$

where *λ* is a hyperparameter to balance clustering loss and regularization term.

### 2.3. Localized SimpleMKKM

Unlike the existing min_*η*_  − min_*H*_ paradigm, SimpleMKKM adopts min_*η*_  − max_*H*_ optimization [20]. However, it is extended to make full use of the information between local sample neighbors and min_*η*_  − max_*H*_ optimization to enhance the clustering effect with a fusion algorithm called localized SimpleMKKM. The objective value of LI-SimpleMKKM can be represented as follows:

(6) $$\begin{array}{c} \begin{array}{c} \min_{\eta }\max_{H}Tr\left(H^{⊤}\overset{n}{\underset{i=1}{\sum }}\left(B^{\left(i\right)}K_{\eta }B^{\left(i\right)}\right)H\right)s.t.\;H\in R^{n\times k},\\ H^{⊤}H=I_{k},\\ \begin{array}{c} \overset{m}{\underset{p=1}{\sum }}\eta_{p}=1,\eta_{p}\geq 0,\forall p, \end{array} \end{array} \end{array}$$

where ∑_*p*=1_^*m*^*η*_*p*_^2^**K**_*p*_ and **B**^(*i*)^=**N**^(*i*)^**N**^(*i*)^^⊤^ with **N**^(*i*)^ ∈ 0,1^*n*×round(*τ* × *n*)^ are the *i*th sample's neighborhood mask matrices; that is, only the samples closest to the target sample will be aligned. This new method is hard to solve with a simple two-step alternating optimization convergence method. To solve this problem, LI-SimpleMKKM first optimizes **H** by a method similar to MKKM and then converts the problem into a problem of finding the minimum with respect to *η*. With proving the differentiability of the minimized formula, the gradient descent method can be used to optimize *η* [21].

## 3. Localized Simple Multiple Kernel K-Means with Matrix-Induced Regularization

According to Liu et al. [21], the relative value of *η*_*p*_ is only dependent on **K**_*p*_, **H**, and **K**_*u*_, where *u* is the largest component of *η*. Only the weights of different kernels are linked, indicating that the LI-SimpleMKKM algorithm is not fully considered the interaction of the kernels when optimizing the kernel weights. This motivates us to derive a regularization term which can measure the correlation between the base kernels to improve this shortcoming.

### 3.1. Formulation

Although the performance of clustering can be improved to some extent by aligning samples with closer samples, there is still room for further improvement of that algorithm.

To address this issue, we define a criterion *ℳ*(**K**_*p*_, **K**_*q*_) to measure the correlation between **K**_*p*_ and **K**_*q*_. A larger *ℳ*(**K**_*p*_, **K**_*q*_) means high correlation between **K**_*p*_ and **K**_*q*_, and a smaller one implies that their correlation is low. We propose to add a matrix-induced regularization *η*^*T*^**M***η* based on LI-SimpleMKKM to improve the shortcomings, enhancing the kernel alignment between multiple kernels and reducing the redundancy of kernels with higher correlation. By fusing the regular term with (6), we can get the objective function as follows:

(7) $$\begin{array}{c} \min_{\eta \in ∆}\max_{H\in R^{n\times k}}Tr\left(H^{⊤}\overset{n}{\underset{i=1}{\sum }}\left(B^{\left(i\right)}K_{\eta }B^{\left(i\right)}\right)H\right)+\frac{\lambda }{2}\eta^{⊤}M\eta \text{,}\\ s.t.\;H\in R^{n\times k}\text{,}\\ H^{⊤}H=I_{k}\text{,}\\ \overset{m}{\underset{p=1}{\sum }}\eta_{p}=1,\eta_{p}\geq 0,\forall p\text{,} \end{array}$$

where *λ* is a trade-off parameter to balance the loss of clustering problem and the regularization term on kernel weights. The regularization term has many types, such as KL divergence and Hilbert–Schmidt independent criterion.

In our proposed algorithm, we set **M**_*pq*_=Tr(**K**_*p*_**K**_*q*_) for each element in **M** to measure the correlation between **K**_*p*_ and **K**_*q*_. Choosing this method makes the calculation not too complicated and adopts the Hilbert–Schmidt independent criterion in disguise, which can reflect the correlation between different base kernels to a certain extent.

The incorporation of *η*^*T*^**M***η* use of the basic kernel better, thus improving clustering performance. Moreover, we can clearly see that if we set *λ*=0, equation (7) is a special case of LI-SimpleMKKM.

Li et al. [22] use *η*^⊤^**M**^(*i*)^*η* instead of *η*^⊤^**M***η* as a regular term, where **M**^(*i*)^ means a matrix with **M**_*pq*_^(*i*)^=*Tr*(**K**_*p*_^(*i*)^**K**_*q*_^(*i*)^), **K**_*η*_^(*i*)^=**N**^(*i*)^^⊤^**K**_*η*_**N**^(*i*)^. Although this method shows excellent performance, we find that the induced regularization of matrices should be global rather than local because the kernel alignment should be for the global kernel matrix. It can also be found from the experimental results in Table 1 that the global kernel-induced regularization has a better effect.

### 3.2. Alternate Optimization

We design a two-step alternating optimization to solve the formula in (7).

(i) Optimizing **H** by *η* is fixed: fixed *η*, the optimization value with respect to **H** in (7) is represented as follows:
  (8) $$\begin{array}{c} \begin{array}{c} \begin{array}{c} \max_{H\in R^{n\times k}}Tr\left(H^{⊤}\overset{n}{\underset{i=1}{\sum }}\left(B^{\left(i\right)}K_{\eta }B^{\left(i\right)}\right)H\right)s.t.\;H^{⊤}H=I_{k}\text{.} \end{array} \end{array} \end{array}$$
Treating the summation (**B**^(*i*)^**K**_*η*_**B**^(*i*)^) as a whole, (8) can be solved by solving for the eigenvalues of the matrix.
(ii) Optimizing *η* by **H** is fixed: fixed **H**, the optimization value with respect to *η* in (7) can be represented as follows:
  (9) $$\begin{array}{c} \begin{array}{c} \begin{array}{c} J\left(\eta \right)=\max_{\eta }Tr\left(H^{⊤}\overset{n}{\underset{i=1}{\sum }}\left(B^{\left(i\right)}K_{\eta }B^{\left(i\right)}\right)H\right)\text{.}+\;\frac{\lambda }{2}\eta^{T}M\eta \;s.t.\;H^{⊤}H=\text{.} \end{array} \end{array} \end{array}$$

We first prove the differentiability of (9), then calculate the gradient, and optimize *η* by the gradient descent method. The first part of the objective function in (9) is as follows:

(10) $$\begin{array}{c} \begin{array}{c} \begin{array}{c} Tr\left(H^{⊤}\overset{n}{\underset{i=1}{\sum }}\left(B^{\left(i\right)}K_{\eta }B^{\left(i\right)}\right)H\right) \end{array} \end{array}\text{.} \end{array}$$

With the hyperparameter *τ* defined, we can regard **B**^(*i*)^**K**_*η*_**B**^(*i*)^ as a whole, which is global kernel alignment and PSD [21]. For convenience, we let **B**^(*i*)^**K**_*η*_**B**^(*i*)^=**T**_*η*_^(*i*)^.

Thus, the function in (9) can be represented as follows:

(11) $$\begin{array}{c} \begin{array}{c} \min_{\eta \in ∆}J\left(\eta \right), \end{array} \end{array}$$

with

(12) $$\begin{array}{c} \begin{array}{c} \begin{array}{c} J\left(\eta \right)=\max_{H}Tr\left(H^{⊤}T_{\eta }^{\left(i\right)}H\right)+\frac{\lambda }{2}\eta^{⊤}M\eta \;s.t.\;H\in R^{n\times k},\\ H^{⊤}H=I_{k}. \end{array} \end{array} \end{array}$$

**Theorem 1 .**

*𝒥*(*η*) in (12) is differentiable. (*∂𝒥*(*η*)/*∂η*_*p*_)=2*η*_*p*_*Tr*(**H**^*∗*^^⊤^**T**_*p*_^(*i*)^**H**^*∗*^)+*λ*∑_*q*=1_^*m*^*η*_*q*_*M*_*pq*_,

where $H^{*}=\left\{\underset{H}{\mathrm{argmax}}\;Tr\left(H^{⊤}T_{\eta }^{\left(i\right)}H\right)s.t.H^{⊤}H=I_{k}\right\}$.

**Proof**

For any given *η* ∈ ∆, the maximum of optimization problem *Tr*(**H**^⊤^**K**_*η*_**H**)*s*.*t*.**H**^⊤^**H**=**I**_*k*_ is unique [21], with $\tilde{H^{*}}\in \left\{\tilde{H^{*}}\left|\tilde{H^{*}}\right.={H^{*}U,{UU}^{⊤}=U^{⊤}U=I}_{k}\right\}$ the corresponding maximizer. According to theorem in [23], the former part of *𝒥*(*η*) is differentiable. By defining other elements in *η* except for *p* as *s* and the latter part of the *𝒥*(*η*) as *𝒥*_2_(*η*), the differential of *𝒥*_2_(*η*)=(*λ*/2)*η*^⊤^**M***η* can be expressed as follows:

(13) $$\begin{array}{c} J_{2}\left(\eta \right)=\frac{\lambda }{2}\left[\begin{array}{cc} \eta_{p} & \eta_{s} \end{array}\right]\left[\begin{array}{cc} M_{pp} & M_{ps}\\ M_{ps} & M_{ss} \end{array}\right]\left[\begin{array}{c} \eta_{p}\\ \eta_{s} \end{array}\right]=\frac{\lambda }{2}\left(\eta_{p}^{2}M_{pp}+2\eta_{p}\eta_{s}M_{ps}+\eta_{s}^{2}M_{ss}\right)\text{,}\\ \frac{\partial J_{2}\left(\eta \right)}{\partial \eta_{p}}=\lambda \eta_{p}M_{pp}+\lambda \eta_{s}M_{ps}\text{,} \end{array}$$

where *p* means one of the components of *η* and *s* means all of the other components so that (*∂𝒥*_2_(*η*)/*∂η*_*p*_)=*λ*∑_*q*=1_^*m*^*η*_*q*_*M*_*pq*_, and the whole *𝒥*(*η*) in (12) is differentiable.

We can solve this problem by designing a gradient descent method. After obtaining the gradient of *𝒥*(*η*) under the premise of satisfying the equality constraints ∑_*p*=1_^*m*^*η*_*p*_=1 and nonnegativity constraints *η*_*p*_ ≥ 0 of *η*, we update *η* by gradient descent [23]. To implement this method, we let *η*_*u*_ become a nonzero unit in *η* and ∇*𝒥*(*η*) indicates the reduced gradient of *𝒥*(*η*). The *p*th (1 ≤ *p* ≤ *m*) element of ∇*𝒥*(*η*) is presented as follows:

(14) $$\begin{array}{c} \begin{array}{c} {\left[\nabla J\left(\eta \right)\right]}_{p}=\frac{\partial J\left(\eta \right)}{\partial \eta_{p}}-\frac{\partial J\left(\eta \right)}{\partial \eta_{u}}\forall p\neq u, \end{array} \end{array}$$

and

(15) $$\begin{array}{c} \begin{array}{c} \begin{array}{c} {\left[\nabla J\left(\eta \right)\right]}_{u}=-\overset{m}{\underset{p=1,p\neq u}{\sum }}{\left[\nabla J\left(\eta \right)\right]}_{p}\text{.} \end{array} \end{array} \end{array}$$

To improve numerical stability, we choose *u* as the largest unit in the vector *η*. The nonnegativity constraint of *η* also needs to be considered during gradient descent.

To minimize *𝒥*(*η*), we define −∇*𝒥*(*η*) as a descent direction. However, if there is an index *p* corresponding to *η*_*p*_=0, with [∇*𝒥*(*η*)]_*p*_ ≥ 0, the situation of *η*_*p*_ < 0 may occur when the gradient is updated, violating the nonnegativity constraint. Under these circumstances, the descent direction for that unit *p* is set as zero. This makes *η* when the gradient is updated as follows:

(16) $$\begin{array}{c} d_{p}=\left\{\begin{array}{cc} 0\text{,} & if\;\eta_{p}=0\;and\;{\left[\nabla J\left(\eta \right)\right]}_{p}\geq 0\text{,}\\ -{\left[\nabla J\left(\eta \right)\right]}_{p}\text{,} & if\;\eta_{p}\geq 0\;and\;p\neq u\text{,}\\ -{\left[\nabla J\left(\eta \right)\right]}_{u}\text{,} & for\;p=u\text{.} \end{array}\right. \end{array}$$

The gradient update adopts the formula *η* ← *η*+*γ ***d**, where *γ* is the step size. We determine the step size *γ* by a one-dimensional linear search method, rather than setting it directly, and in order to ensure global convergence, this method has appropriate stopping criteria, for example, Armijo's rule [21].

The specific calculation steps of the algorithm in equation (13) are detailed in Algorithm 1.

**Theorem 2 .**

The proposed algorithm is converged.

**Proof**

Note that for the *k*th iteration, Tr(**H**^⊤^**T**_*η*_^(*i*)^**H**) will be bigger than *k* + 1th iteration. In each iteration, the gradient of *η*_*p*_ is smaller than 0 by equation (14) because *u* is the component of *η*, and in order to get the maximum of Tr(**H**^⊤^**T**_*η*_^(*i*)^**H**), Tr(*η*_*u*_**H**^⊤^**T**_*u*_^(*i*)^**H**) should be larger than other parts, so the differential of it is bigger than others. The component *u* has the gradient which is the opposite number of other component gradients' sum by the equation (15). According to the equation (16), the component of *p* will be bigger, while the coefficient of *u* will be smaller, and we can let ∆ as the difference of the *k*th iteration and *k* + 1th's, with ∆=Tr*γ*(*d*_1_**H**^⊤^**T**_1_^(*i*)^**H**)+Tr*γ*(*d*_2_**H**^⊤^**T**_2_^(*i*)^**H**)+…+Tr*γ*(*d*_*m*_**H**^⊤^**T**_*m*_^(*i*)^**H**), ∑_*p*=1,*p*≠*u*_^*m*^*d*_*p*_ with **H**^⊤^**T**_*u*_^(*i*)^**H** as the largest part of each **H**^⊤^**T**^(*i*)^**H**, *γ* is bigger than 0, it can be easy to get the conclusion ∆ is smaller than 0, because the non-negativity of *η ***a**nd kernel matrix, the former term has the lower bound 0 and convex, so the former term's convergence is been proofed.

As for the latter term (*λ*/2)*η*^⊤^**M***η* with the similar thought, it is also decreasing monotonically because **M** is a PSD matrix, *η* is not negative, and *λ* is bigger than 0; the second derivative of *𝒥*_2_(*η*) can be easy to be calculated bigger than 0 (since each element of **M** is bigger than 0), so the latter term has the lower bound 0 and convex. At the same time, the whole equation (13) is monotonically decreasing and lower-bounded.

### 3.3. Computational Complexity Analysis

We theoretically analyze the time complexity of the algorithm LI-SimpleMKKM-MR. We assume that *n* and *m* denote the number of samples and the number of base kernels. LI-SimpleMKKM-MR based on Algorithm 1 first computes a neighborhood mask matrix with computational complexity *𝒪*(*n*^2^ log_2_ *n*) and then computes the regularization term with computational complexity *𝒪*(*m*^3^). Therefore, the time complexity of LI-SimpleMKKM-MR is (*n*^3^+*n*^2^ log_2_ *n*+*m*^3^) per iteration.

Let us compare the complexity of LI-SimpleMKKM-MR and LI-SimpleMKKM. Since in most cases, the number of base kernels is much fewer than the number of samples (*m* ≪ *n*), compared with LI-SimpleMKKM (*n*^3^), the time complexity of the proposed method does not increase significantly.

## 4. Experiments

### 4.1. Datasets

In this section, we evaluate the clustering performance of our algorithm on a set of standard MKKM benchmark datasets, including Oxford Flower17(FLO17), Flower102(FLO102) (https://www.robots.ox.ac.uk/~+vgg/data/flowers/), Protein Fold Prediction(proteinFold) (https://mkl.ucsd.edu/dataset/protein-fold-prediction/), Digital (https://ss.sysu.edu.cn/~+py/), Caltech101-25views(Cal-25views), and Caltech101-7classes(Cal-7classes) (https://files.is.tue.mpg.de/pgehler/projects/iccv09/). Caltech101-25views refers to the number of kernels randomly selected by 25, and Caltech101-7classes refers to the number of classes randomly selected by 7. The details of these can be found in Table 2. We can compare the performance of the different MKKM algorithms using these datasets.

### 4.2. Compared Algorithms

In addition to the localized SimpleMKKM with matrix-induced regularization, we tested nine other comparative algorithms from the other MKKM algorithms, including, average kernel k-means (**Avg-KKM**), multiple kernel k-means (**MKKM**) [10], localized multiple kernel k-mean (**LMKKM**) [12], optimal neighborhood kernel clustering (**ONKC**) [24], multiple kernel k-mean with matrix-induced regularization (**MKKM-MR**) [14], multiple kernel clustering with local alignment maximization (**LKAM**) [22], multiview clustering via late fusion alignment maximization (**LF-MVC**) [25], simple multiple kernel k-means (**SimpleMKKM**) [20], and localized SimpleMKKM (**LI-SimpleMKKM**) [21].

The implementations of the comparison algorithms are publicly available in the corresponding papers, and we directly apply them to our experiments without tuning. Among the previous algorithms, ONKC, MKKM-MR, LKAM, LF-MVC, and LI-SimpleMKKM need to adjust hyperparameters. Based on the published papers and actual experimental results, we show the best clustering results of the previous methods by tuning the hyperparameters on each dataset.

### 4.3. Experimental Settings

In all experiments, to reduce the difference between different views, all the base kernels are first centered and then scaled so that for all *i* and *p*, we have **K**_*p*_(**x**_*i*_, **x**_*i*_)=1. For our proposed algorithm, its trade-off parameters *λ* and *τ* are chosen from [2^−15^, 2^−13^,…, 2^10^] and [0.05,0.1,…, 0.95] × *n* by grid search, where *n* is the number of samples.

For all the datasets, we set the number of clusters *k* according to the actual number of categories in the dataset. We engage 4 indicators: clustering accuracy (ACC), normalized mutual information (NMI), purity, and rand index to measure the effect of clustering. To reduce the harmful effects of randomness, we initialized and executed all algorithms fifty times (50×) to obtain the mean and variance of the experimental indicators.

### 4.4. Experimental Results

Table 3 reports the ACC, NMI, purity, and rand index of the previously mentioned algorithms on all 6 datasets. The following observations were made based on the results:

The proposed localized SimpleMKKM with matrix-induced regularization significantly outperforms localized SimpleMKKM. For example, it outperforms the LI-SimpleMKKM algorithm by 1.8%, 0.1%, 3.1%, 0.3%, 0.6%, and 3.4% in terms of ACC on Flower17, Flower102, ProteinFold, DIGIT, Caltech-25 views, and Caltech-7 classes datasets. These results validate the effectiveness of enhancing the correlation between matrices.

Also, our proposed LI-SimpleMKKM-MR significantly outperforms the MKKM-MR algorithms by 3.6%, 3.8%, 4.7%, 7.5%, 3.3%, and 6.3% in terms of ACC on Flower17, Flower102, ProteinFold, DIGIT, Caltech-25 views, and Caltech-7 classes datasets. This result proves that utilizing the data's local structure and min_*η*_  − max_*H*_ optimization improves the clustering effect very well.

The proposed algorithm adopts the advanced formulation and uses matrix-induced regularization to improve the correlation between kernel matrices, reducing redundancy and increasing the diversity of selected kernel matrices, making it superior to its counterpart.

Together, these factors make LI-SimpleMKKM-MR significantly improved over other algorithms on the same dataset. In addition, due to time complexity and memory constraints, the effect of LMKKM on some datasets has not been shown.

### 4.5. Parameter Sensitivity of LI-SimpleMKKM-MR

We designed comparative experiments to study the influence of the setting of two hyperparameters, localized alignment, and matrix-induced regularization, on the clustering effect. According to equation (7), LI-SimpleMKKM-MR tunes the clustering performance by setting two hyperparameters *λ* and *τ*, referring to the regularization balance factor and the nearest neighbor ratio.

We experimentally show the difference in clustering performance in *λ* and *τ* in all benchmark datasets.

**Figure 1:**
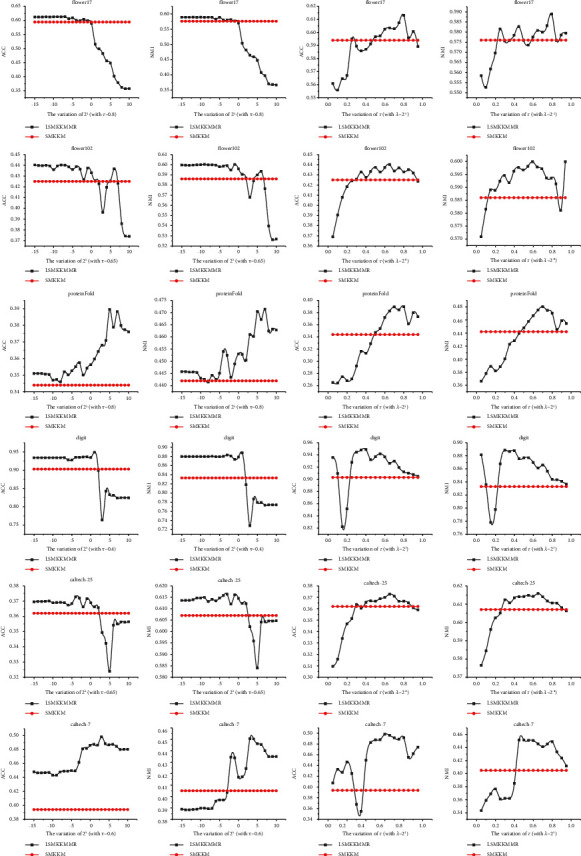
Sensitivity of the proposed method LI-SimpleMKKM-MR with a variation of *λ* and *τ* compared with SimpleMKKM.

Figure 1 shows the ACC and NMI of our algorithm by varying one of *τ* or *λ* with the other one fixed. Based on these figures, we can conclude that (1) as the value of *τ* increases, the ACC and NMI of each dataset increase to their highest value and, correspondingly, decrease when *τ* decreases and (2) by keeping the *τ* unchanged, the ACC and NMI will exceed SimpleMKKM and be steady when *λ* is small.

Hence, we conclude that our proposed algorithm presents a new state-of-the-art performance for clustering compared to other algorithms that only preserve the global kernel, such as LI-MKKM. Thus, it focuses on preserving the local structure of the data as specific results are displayed in Table 1.

On top of min_*η*_  − max_*H*_ optimization, the clustering performance improves when the parameters are appropriately set by combining matrix-induced regularization and local alignment.

### 4.6. Convergence of LI-SimpleMKKM-MR

**Figure 2:**
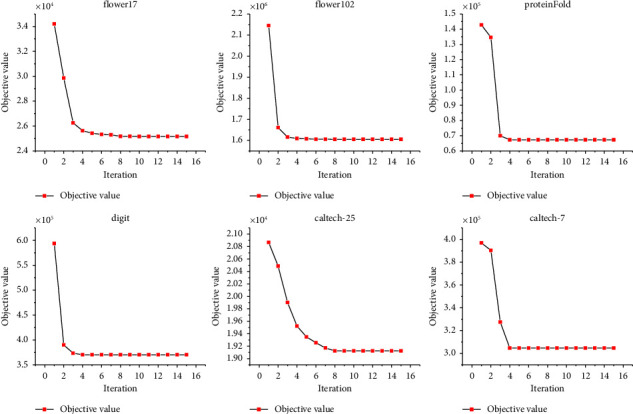
Proposed algorithm convergence illustration on flower17, flower102, ProteinFold, digit, Caltech-25, and Caltech-7 datasets.

In addition to theoretical verification, we experimentally verify the convergence of the algorithm. We present simulations of our proposed algorithm using different datasets in Figure 2. According to the results, the object value of the proposed algorithm oscillates first, then decreases monotonically, and finally converges in several iterations. Moreover, we know from experiments that most datasets can converge in fewer than 10 iterations. This result is comparable to the state-of-the-art methods.

### 4.7. Performance of LI-SIMPLEMKKM-MR by Learned H

**Figure 3:**
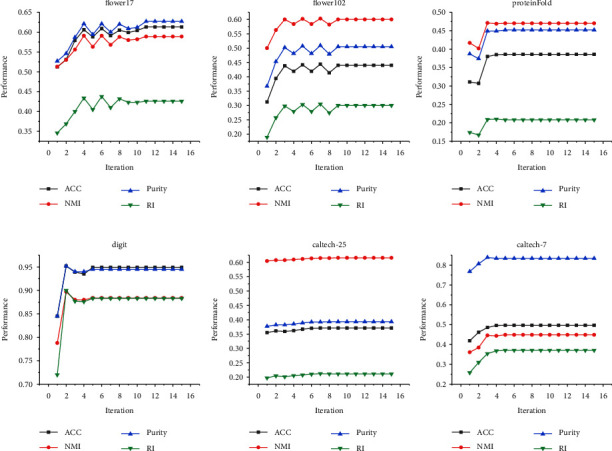
Clustering performance of **H** iteratively in LI-SimpleMKKM-MR learning on flower17, flower102, ProteinFold, digit, Caltech-25, and Caltech-7 datasets.

We calculate the 4 clustering metrics at each iteration to show the variety of clustering performance variations of the learned **H** in different datasets and plot them in Figure 3. As observed, the clustering performance increased firstly with iterations and remained stable after oscillation.

### 4.8. Running Time of LI-SimpleMKKM-MR

**Figure 4:**
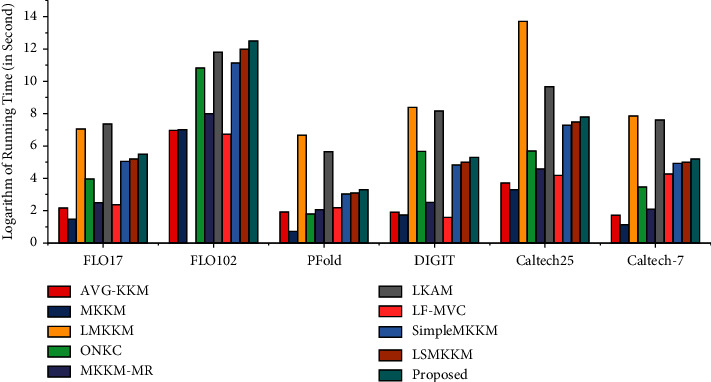
Running time comparison of different algorithms on all benchmark datasets (base 2 logarithm in seconds). The experimental environment is a desktop with Ubuntu 20.0 OS, Intel Core-i7-9700K cpu @ 3.60 GHz, 94.2 G RAM.

We report the running time comparison of all the baseline algorithms and LI-SimpleMKKM-MR on different datasets in Figure 4. With the analysis of the time complexity in Section 3.3 and the experiment result from Figure 4, even though there are additional computational steps, we found that LI-SimpleMKKM-MR does not significantly increase in computation time.

## 5. Conclusion

Although LI-SimpleMKKM can address the task of multiple kernel k-means in a min_*η*_  − max_*H*_ optimization and realize the local alignment, it does not sufficiently account for the correlation between the basis kernels. This work proposes an LI-SimpleMKKM-MR algorithm that combines the sample localized alignment and matrix-induced regularization to solve this problem. Theoretically and experimentally, our method has demonstrated the best performance in clustering optimization and outperforms existing algorithms. In further research, we will apply this algorithm to incomplete MKKM problems.

## Figures and Tables

**Algorithm 1:**
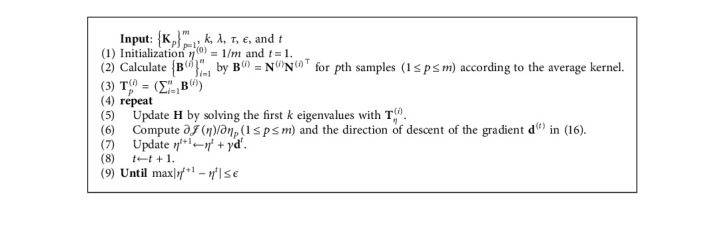
LI-SimpleMKKM-MR.

**Table 1:** Comparation of localized **M** and global **M** in ACC.

Datasets	Localized **M**	Global **M**
Flower17	51.8 ± 1.0	61.3 ± 1.5
Flower102	37.4 ± 0.8	44.0 ± 1.0
ProteinFold	36.4 ± 1.5	39.0 ± 1.4
Digital	83.4 ± 1.2	94.9 ± 3.0
Cal-25views	36.6 ± 0.7	37.3 ± 1.1
Cal-7classes	47.8 ± 1.0	49.8 ± 0.8

**Table 2:** Specification of our 6 benchmark datasets.

Datasets	#Samples	#Kernels	#Classes
Flower17	1360	7	17
Flower102	8189	4	102
ProteinFold	694	12	27
Digital	2000	3	10
Cal-25views	1530	25	102
Cal-7classes	1474	6	7

**Table 3:** ACC, NMI, purity, and Rand index data of localized-SimpleMKKM-matrix-induced regularization with nine comparison methods on six benchmark datasets.

Dataset	AVG-KKM	MKKM [10]	LMKKM [12]	ONKC [24]	MKKM-MR [14]	LKAM [22]	LF-MVC [25]	SimpleMKKM [20]	LI-SimpleMKKM [21]	Proposed
*ACC (%)*										
FLO17	51.3 ± 1.4	43.6 ± 1.7	42.7 ± 1.7	43.4 ± 2.0	57.7 ± 1.2	48.9 ± 0.9	56.7 ± 1.5	59.4 ± 1.5	59.1 ± 1.1	**61.3** **±** **1.5**
FLO102	27.1 ± 0.8	22.4 ± 0.8	—	39.5 ± 0.7	40.2 ± 0.9	41.4 ± 0.8	38.4 ± 1.2	42.5 ± 0.8	44.0 ± 1.0	**44.1** **±** **1.0**
PFold	29.1 ± 1.4	27.1 ± 1.0	22.4 ± 0.7	35.4 ± 1.5	34.3 ± 1.6	34.2 ± 1.6	33.3 ± 2.0	34.4 ± 1.9	35.9 ± 1.5	**39.0** **±** **1.4**
DIGIT	88.8 ± 0.1	47.2 ± 0.6	47.2 ± 0.7	89.5 ± 0.1	87.4 ± 0.1	**95.0** **±** **0.3**	89.2 ± 0.1	90.3 ± 0.1	94.6 ± 0.1	94.9 ± 3.0
Cal-25	34.2 ± 1.0	32.8 ± 0.1	22.6 ± 0.7	34.3 ± 1.2	34.0 ± 1.2	37.2 ± 1.1	34.6 ± 1.1	36.2 ± 1.2	36.7 ± 0.9	**37.3** **±** **1.1**
Cal-7	33.7 ± 0.1	33.3 ± 0.2	34.4 ± 0.1	46.0 ± 4.0	43.5 ± 3.9	49.4 ± 1.0	42.3 ± 2.7	39.4 ± 1.5	46.4 ± 0.9	**49.8** **±** **0.8**

*NMI (%)*										
FLO17	49.9 ± 0.9	44.3 ± 1.3	43.8 ± 1.1	42.9 ± 1.3	56.1 ± 0.7	48.1 ± 0.6	54.6 ± 1.0	57.6 ± 0.1	57.7 ± 0.5	**58.9** **±** **1.0**
FLO102	46.0 ± 0.5	42.7 ± 0.2	—	56.1 ± 0.4	56.7 ± 0.5	56.9 ± 0.3	54.9 ± 0.4	58.6 ± 0.5	60.0 ± 0.4	**60.1** **±** **0.4**
PFold	40.3 ± 1.2	38.1 ± 0.6	34.8 ± 0.6	44.1 ± 0.8	43.2 ± 1.1	43.7 ± 1.0	42.3 ± 1.2	44.2 ± 1.2	45.2 ± 1.3	**48.0** **±** **0.9**
DIGIT	80.7 ± 0.2	48.7 ± 0.7	48.3 ± 0.2	81.7 ± 0.1	79.5 ± 0.1	89.4 ± 0.1	81.2 ± 0.2	83.3 ± 0.1	90.0 ± 0.1	**90.3** **±** **1.6**
Cal-25	59.7 ± 0.5	58.6 ± 0.5	51.9 ± 0.3	59.6 ± 0.8	59.3 ± 0.5	**62.0** **±** **0.6**	59.5 ± 0.7	60.7 ± 0.5	61.4 ± 0.4	61.5 ± 0.5
Cal-7	34.9 ± 0.3	30.0 ± 0.3	30.7 ± 0.1	40.0 ± 1.0	43.1 ± 0.8	43.3 ± 0.2	40.0 ± 0.3	40.5 ± 0.4	39.2 ± 1.3	**44.9** **±** **0.1**

*Purity (%)*										
FLO17	52.3 ± 1.2	45.1 ± 1.4	44.6 ± 1.5	45.1 ± 1.8	59.2 ± 1.1	50.1 ± 0.6	57.5 ± 1.6	60.5 ± 1.3	59.7 ± 0.3	**62.7** **±** **1.6**
FLO102	32.3 ± 0.6	27.8 ± 0.4	—	45.6 ± 0.7	46.3 ± 0.8	48.0 ± 0.6	44.6 ± 0.8	48.6 ± 0.7	50.3 ± 0.7	**50.5** **±** **0.7**
PFold	37.3 ± 1.6	33.7 ± 0.9	31.1 ± 1.0	42.0 ± 1.2	41.2 ± 1.4	41.6 ± 1.3	40.6 ± 1.6	41.4 ± 1.6	42.5 ± 1.6	**46.2** **±** **1.5**
DIGIT	88.8 ± 0.1	50.1 ± 0.7	50.2 ± 0.3	89.5 ± 0.1	87.4 ± 0.1	**95.0** **±** **0.3**	89.2 ± 0.1	90.3 ± 0.1	94.6 ± 0.1	94.9 ± 2.0
Cal-25	36.2 ± 1.0	34.9 ± 0.1	24.4 ± 0.6	36.6 ± 1.1	36.1 ± 1.0	**39.4** **±** **1.1**	36.8 ± 1.0	38.2 ± 1.1	39.1 ± 0.8	39.3 ± 1.1
Cal-7	79.0 ± 0.2	76.7 ± 0.2	74.9 ± 0.1	81.2 ± 0.9	82.9 ± 0.4	83.2 ± 0.2	81.6 ± 0.3	83.3 ± 0.3	80.8 ± 0.7	**83.4** **±** **0.1**

*Rand Index (%)*										
FLO17	32.2 ± 1.3	26.3 ± 1.3	20.6 ± 1.1	35.2 ± 1.5	39.9 ± 1.3	31.6 ± 0.8	44.1 ± 0.4	41.5 ± 1.1	40.9 ± 0.8	**42.6** **±** **1.4**
FLO102	15.5 ± 0.5	12.1 ± 0.5	—	24.9 ± 0.5	25.5 ± 0.6	27.2 ± 0.6	25.5 ± 1.0	28.5 ± 0.8	29.9 ± 0.8	**30.0** **±** **0.8**
PFold	14.4 ± 1.8	12.1 ± 0.7	7.8 ± 0.4	18.0 ± 1.1	17.2 ± 1.5	20.1 ± 1.1	16.5 ± 2.0	17.6 ± 1.9	19.8 ± 1.2	**20.8** **±** **1.5**
DIGIT	77.4 ± 0.2	31.4 ± 0.6	31.3 ± 0.2	81.7 ± 0.3	81.3 ± 0.1	**90.8** **±** **2.3**	78.0 ± 0.1	80.6 ± 0.2	88.2 ± 0.1	88.3 ± 2.9
Cal-25	18.5 ± 0.9	17.3 ± 0.1	8.3 ± 0.6	18.6 ± 1.2	18.4 ± 0.8	**21.5** **±** **1.0**	18.9 ± 0.9	20.4 ± 0.1	20.1 ± 0.7	21.1 ± 0.8
Cal-7	26.5 ± 0.3	23.5 ± 0.3	20.3 ± 0.1	30.9 ± 1.7	31.5 ± 2.2	36.6 ± 0.5	29.3 ± 0.8	30.3 ± 0.7	32.2 ± 1.3	**37.1** **±** **0.3**

Bold indicates better results in comparison with other algorithms.

## Data Availability

The data that support the findings of this study are openly available at https://www.robots.ox.ac.uk/~+vgg/data/flowers/, https://mkl.ucsd.edu/dataset/protein-fold-prediction/, https://ss.sysu.edu.cn/~+py/, and https://files.is.tue.mpg.de/pgehler/projects/iccv09/.

## References

[1] Pehlivan N. Y., Turksen I. B. (2021). A novel multiplicative fuzzy regression function with a multiplicative fuzzy clustering algorithm. *Romanian Journal of Information Science and Technology*.

[2] Pozna C., Precup R.-E., Horváth E., Petriu E. M. (2022). Hybrid particle filter–particle swarm optimization algorithm and application to fuzzy controlled servo systems. *IEEE Transactions on Fuzzy Systems*.

[3] Borlea I.-D., Precup R.-E., Borlea A.-B. (2022). Improvement of K-means cluster quality by post processing resulted clusters. *Procedia Computer Science*.

[4] Ieracitano C., Paviglianiti A., Mammone N., Versaci M., Pasero E., Morabito F. C. (2020). Socnnet: an optimized sobel filter based convolutional neural network for sem images classification of nanomaterials. *Progresses in Artificial Intelligence and Neural Systems*.

[5] Zhang J., Ma Z. (2020). Hybrid fuzzy clustering method based on FCM and enhanced logarithmical PSO (ELPSO). *Computational Intelligence and Neuroscience*.

[6] De Paris R., Quevedo C. V., Ruiz D. D., Norberto de Souza O., Barros R. C. (2015). Clustering molecular dynamics trajectories for optimizing docking experiments. *Computational Intelligence and Neuroscience*.

[7] Gao W. (2016). Improved ant colony clustering algorithm and its performance study. *Computational Intelligence and Neuroscience*.

[8] Zhou J., Wang D., Ling L., Li M., Lai K.-W. (2022). Comparative analysis of the performance of complex texture clustering driven by computational intelligence methods using multiple clustering models. *Computational Intelligence and Neuroscience*.

[9] Qian G., Wu Y., Ferrari D., Qiao P., Hollande F. (2016). Semisupervised clustering by iterative partition and regression with neuroscience applications. *Computational Intelligence and Neuroscience*.

[10] Zhao B., Kwok J. T., Zhang C. Multiple kernel clustering.

[11] Huang H.-C., Chuang Y.-Y., Chen C. S. (2011). Multiple kernel fuzzy clustering. *IEEE Transactions on Fuzzy Systems*.

[12] Gönen M., Margolin A. A. (2014). Localized data fusion for kernel k-means clustering with application to cancer biology. *Advances in Neural Information Processing Systems*.

[13] Du L., Zhou P., Shi L. Robust multiple kernel k-means using l21-norm.

[14] Liu X., Dou Y., Yin J., Wang L., Zhu E. Multiple kernel k-means clustering with matrix-induced regularization.

[15] Wang S., Zhu E., Hu J. (2019). Efficient multiple kernel k-means clustering with late fusion. *IEEE Access*.

[16] Liu X., Zhu X., Li M. Efficient and effective incomplete multi-view clustering.

[17] Lan Z.-z., Bao L., Yu S.-I., Liu W., Hauptmann A. G. Double fusion for multimedia event detection.

[18] Houthuys L., Langone R., Suykens J. A. (2018). Multi-view kernel spectral clustering. *Information Fusion*.

[19] Tao Z., Liu H., Li S., Ding Z., Fu Y. From ensemble clustering to multi-view clustering.

[20] Liu X. (2022). Simplemkkm: simple multiple kernel k-means. *IEEE Transactions on Pattern Analysis and Machine Intelligence*.

[21] Liu X., Zhou S., Liu L. Localized simple multiple kernel k-means.

[22] Li M., Liu X., Wang L., Dou Y., Yin J., Zhu E. Multiple kernel clustering with local kernel alignment maximization.

[23] Rakotomamonjy A., Bach F., Canu S., Grandvalet Y. (2008). SimpleMKL. *Journal of Machine Learning Research*.

[24] Liu X., Zhou S., Wang Y. Optimal neighborhood kernel clustering with multiple kernels.

[25] Wang S., Liu X., Zhu E. Multi-view clustering via late fusion alignment maximization.

[26] Qiu J., Xu H., Zhu X. (2020). Localized simple multiple kernel K-means with matrix induced regularization. https://arxiv.org/pdf/2005.04975.pdf.

